# PiDose: an open-source system for accurate and automated oral drug administration to group-housed mice

**DOI:** 10.1038/s41598-020-68477-2

**Published:** 2020-07-14

**Authors:** Cameron L. Woodard, Wissam B. Nasrallah, Bahram V. Samiei, Timothy H. Murphy, Lynn A. Raymond

**Affiliations:** 1 Graduate Program in Neuroscience, University of British Columbia, Vancouver, BC Canada; 2 Department of Psychiatry and Djavad Mowafaghian Centre for Brain Health, University of British Columbia, Vancouver, BC Canada; 3 MD/PhD Program, University of British Columbia, Vancouver, BC Canada; 4 Undergraduate Program in Engineering, University of British Columbia, Vancouver, BC Canada; 5 School of Biomedical Engineering, University of British Columbia, Vancouver, BC Canada; 6 2255 Wesbrook Mall, Detwiller Pavilion Rm. 4834, Vancouver, BC V6T 1Z3 Canada

**Keywords:** Translational research, Drug discovery, Behavioural methods

## Abstract

Drug treatment studies in laboratory mice typically employ manual administration methods such as injection or gavage, which can be time-consuming to perform over long periods and cause substantial stress in animals. These stress responses may mask or enhance treatment effects, increasing the risk of false positive or negative results and decreasing reliability. To address the lack of an automated method for drug treatment in group-housed mice, we have developed PiDose, a home-cage attached device that weighs individual animals and administers a daily dosage of drug solution based on each animal’s bodyweight through their drinking water. Group housed mice are identified through the use of RFID tagging and receive both regular water and drug solution drops by licking at a spout within the PiDose module. This system allows animals to be treated over long periods (weeks to months) in a fully automated fashion, with high accuracy and minimal experimenter interaction. PiDose is low-cost and fully open-source and should prove useful for researchers in both translational and basic research.

## Introduction

Biological research often involves treating experimental rodents with compounds over extended periods. A variety of routes of administration are used in these studies, with the goal to optimize delivery of the agent while reducing the potential for injury and procedure-associated stress. Parenteral administration via subcutaneous or intraperitoneal injection is often used due to the high bioavailability of injected drugs; however, repeated restraint and injection causes stress and puts the animal at risk of physical complications^1,2,3^. These stress responses are particularly undesirable in behavioural studies, as chronic stress affects a variety of behaviours and may mask treatment affects and increase the risk of Type I/II errors^4,5^. An alternative to injection is oral administration, which is often useful in a pre-clinical context as oral drug treatment is the most common and convenient route of administration in humans. Unfortunately, oral gavage presents the same problems as injection regarding treatment stress and the potential for injury^1,6^. To avoid this, several studies have provided methods for the voluntary feeding of drugs to animals in a palatable form (e.g. sucrose water, peanut butter)^7,8,9,10^. This avoids some of the side effects associated with injection and gavage, but is time-consuming for chronic experiments and involves extensive experimenter interaction, which in itself may be enough to increase animal stress^11^. To circumvent the need for manual administration, other studies have mixed the drug with the animal’s drinking water^12,13,14,15^. However, this method typically estimates drug dosage based on the average bodyweight and water consumption for all mice in a cage. This relies on the assumption that mice are drinking an amount of water that is directly proportional to their bodyweight, for which there is not clear support. To avoid this caveat, animals can be single-housed, or double-housed with a divider. Unfortunately, this still does not guarantee consistent dosing over time as water consumption may vary from day to day.

An approach that has recently gained popularity in rodent research is to automate experimental procedures using devices that the animal can freely access from within their home-cage. These systems provide the combined benefits of increasing the throughput of experiments and volume of data that can be collected, while also decreasing experimenter interaction and animal stress. Open-source tools that enable the home-cage monitoring of feeding and drinking^16,17^, bodyweight^18,19^, activity levels^20,21^, and more complex learning tasks^22,23,24,25,26^ have all been published in recent years. In one notable study, the authors developed a proprietary home-cage methodology to automatically dose group-housed mice with the synthetic nucleoside BrdU over several days^27^. This system used an RFID detector to individually identify transponder-tagged mice and dispense drug solution to them through a liquid port. Dosage of drug could be individually specified for different mice, however animals had to be manually weighed in order to set drug dose, and the accuracy of drug delivery was not directly assessed. Aside from this study, the potential for long-term home-cage drug administration in experimental rodents has not been explored.

To address this, we have developed PiDose—an open-source tool for home-cage oral drug administration. PiDose allows mice to freely access a chamber (the ‘dosing module’) from their home-cage where they are automatically weighed and lick a spout to obtain drops of drug solution. This design ensures that mice consistently ingest the drug, as liquid is delivered directly into the mouth in response to licking. Mice are RFID-tagged to discriminate group-housed animals, and the drug volume each mouse receives can be individually customized based on their dosing condition and current bodyweight. Once they have received the required dose of drug for each day, they receive only water from the spout. This system allows for accurate dosing to be maintained over long periods (weeks to months) with minimal experimenter interaction. It is low-cost (~ $300) and built with 3D-printed parts and electronic components that can be easily obtained. We provide here a description and demonstration of PiDose, as well as the software and a list of parts required to build the system.

## Methods

### Hardware

**Figure 1:**
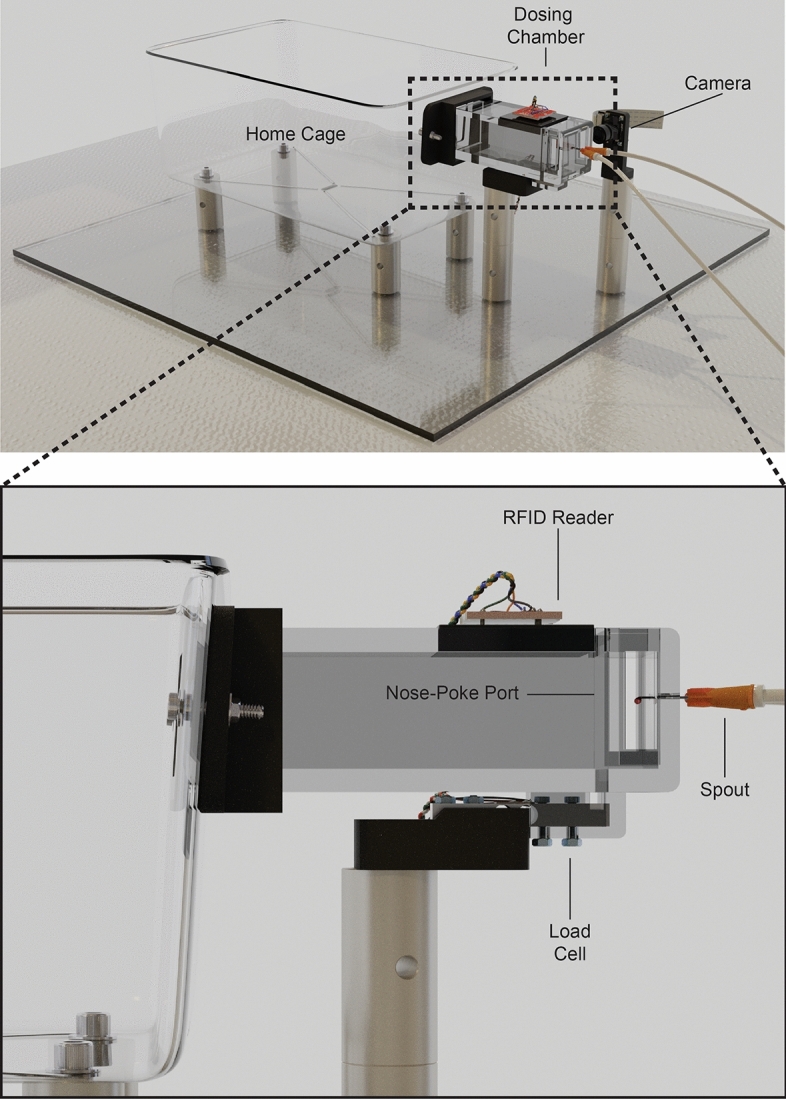
The PiDose system. PiDose consists of a dosing module mounted adjacent to a standard mouse shoebox home-cage. An entranceway allows animals to freely access the dosing module, where they can obtain both water and drug solution drops from a spout. An adjacent camera captures photos of drop delivery. The dosing module is mounted on a load cell which collects bodyweight measurements from mice. Transponder-tagged mice are identified by an RFID reader and can access the spout through a nose-poke port. Drop delivery is triggered by licking at the spout. Renderings by Luis Bolaños.

PiDose consists of a modified mouse home-cage with an opening to allow animals to freely access a linked 3D-printed dosing module (Fig. 1). The module is supported by a free-floating 0.78 kg load cell (Phidgets 3132) mounted on a post that does not contact the cage, similar to the configuration described in Noorshams et al. (2017)^18^. A 3D-printed entranceway is attached to the cage and frames the chamber opening, allowing the mouse to more easily enter the dosing module. At the opposite end of the dosing module from the entrance, a nose-poke port permits access to a spout which dispenses drops from two separate liquid reservoirs. This spout is wired to a capacitive touch sensor controller (Adafruit 1982) to detect individual licks. Adjacent to the nose-poke port, an RFID reader (Sparkfun SEN-11828) is inset into the ceiling of the dosing module to identify transponder-tagged animals as described in Bolaños et al. (2017)^28^. A camera (Waveshare 10299) is positioned to one side of the dosing module in order to capture images of mice during drop delivery. The cage, dosing module, and camera are all attached to aluminum spacers (Siskiyou AS-2.00) mounted on a polycarbonate sheet. Four PiDose cages were built and used to perform the described experiments. The total cost for one system is ~ 300 USD and a full parts list for PiDose can be found in Supplementary Table S1. Design files for 3D-printing can be found in Supplementary Data 1, and full instructions for constructing and wiring a PiDose cage can be found online (https://osf.io/rpyfm/).

In order to dispense liquid from two sources with minimal dead volume and cross-contamination, we constructed a double-spout from two parallel and attached 18G gauge needles. To dispense drops from the double-spout, we use two complementary approaches. Regular water drops are dispensed from a reservoir using a gravity-fed valve-based system, as described previously^22,29^. This method of water delivery is reliable; however, the drops vary in size due to changes in the level of the water reservoir or changes in resistance. As a result, the valve opening time must be recalibrated every few days to ensure consistency. For dispensing drops of drug solution, we use an open-source syringe pump design by Wijnen et al. (2014)^30^ which provides consistent and accurate liquid displacement over long periods. This syringe pump is constructed primarily from 3D-printed parts and uses a NEMA17 stepper motor (Sparkfun ROB-09238) and threaded steel rod to move the plunger of a syringe. Through calibration of these pumps, we determined that 57 steps are required to reliably give a drop of 10 µL from a 30 mL syringe. To assess the accuracy of the syringe pumps, we dispensed one hundred 10 µL drops into a dish and weighed the dispensed water, repeating this 8–10 times for each pump. Full instructions and parts required to construct this syringe pump can be found online (https://hackaday.io/project/27046-open-source-syringe-pump).

### Software and dosing methodology

All PiDose components are connected to and controlled by custom software running on a Raspberry Pi 3B+ micro-computer running the Raspbian operating system. The software controlling the PiDose components and recording data is written in Python 3 and is available online (https://github.com/cameron-woodard/PiDose). The program runs continuously while animals are housed in the cage. When a mouse enters the dosing module, it is detected by the RFID reader and the program loads the relevant parameters and daily stats for the mouse. The size and shape of the chamber ensures that only one mouse can fully enter the dosing module and be detected by the reader at a time, however mice frequently go in and out of the chamber in quick succession. To ensure that the correct mouse is identified, the PiDose program continually monitors a ‘tag-in-range’ logic signal that indicates whether an RFID is currently in range of the reader. Every time a tag goes in and out of range of the reader (even momentarily), a new read of the RFID is triggered to confirm whether it is the same or a different mouse. If the RFID is not recognized by the reader, possibly due to an incomplete read, the program will simply wait until the mouse leaves the chamber and attempt to read their ID again when they re-enter. If this happens too many times, however, a reboot of the Raspberry Pi will be automatically triggered.

For the duration the mouse is in the module, weight readings are collected from the load cell at 5 Hz and the capacitive sensor is activated on the spout. A lick at the spout will trigger the subsequent delivery of a water or drug drop depending on the mouse’s treatment condition, and the number of drops they have received so far that day. Mice receive drug drops starting at midnight until they have received the required number for the day, and then receive water drops for the remainder of the 24-h cycle. Immediately following drop delivery, an image is taken which can be used to validate that the animal has their mouth on the spout and the drop was not delivered in error. A 10-s timeout follows before the mouse can trigger delivery of another drop, in order to ensure that they fully ingest the liquid. After the mouse exits the dosing module, the capacitive trigger is deactivated, weight collection stops, and a 30-s waiting period is triggered. If no mouse is detected before the end of this wait period, a sample of 20 readings is collected from the load-cell. If none of these readings is further than 0.1 g away from the mean, the load-cell is tared.

Based on values collected from the load cell, an average daily weight is calculated for each mouse at midnight by rounding all values collected in the previous 24 h to one decimal point, removing outliers and taking the mode of these values (i.e. the most commonly occurring weight). This is then used to determine the number of drug drops that the mouse receives the following day. All events (e.g. entrance, lick, drop delivery) are recorded to a text file for that mouse with an event code and timestamp. Timestamped weights are recorded into a separate daily weight text file for each mouse, and a new file is created at midnight on each day. A summary file records the daily water and drug drops received, and the weight for the mouse on each day of treatment.

### Animals

A total of 8 wildtype FVB/N mice and 10 YAC128 mice (FVB/N background, line 55) were used in experiments. The YAC128 mouse is a model of Huntington’s disease that uses a yeast artificial chromosome to express the full-length human huntingtin gene with 128 CAG repeats^31^. All animals were male and were housed and treated in groups of 3–4 littermates in a temperature and humidity-controlled room on a 12/12 h light/dark cycle (lights on at 6 AM). All procedures were conducted in accordance with the Canadian Council on Animal Care and approved by the University of British Columbia Committee on Animal Care.

### RFID capsule implantation

To enable identification of group-housed mice, animals were implanted with glass RFID capsules (Sparkfun SEN-09416) prior to treatment as described in Woodard et al. (2017)^22^. Briefly, animals were anesthetized with isoflurane and given buprenorphine via subcutaneous injection (0.05 mg/kg) for analgesia. Betadine was applied to disinfect the incision site, and a small incision was made in the upper thoracic torso. A sterile injector (Fofia ZS006) was then used to insert the RFID capsule subcutaneously below the nape of the neck. The incision was sutured, and the animal was removed from anesthesia, allowed to recover, and then returned to its home-cage. Animals were monitored for the following 3 days to ensure healthy recovery and proper placement of the RFID capsule. Animals were given at minimum one week to fully recover following surgery before being used for any experiments.

### Drug treatment

For drug treatment experiments, memantine hydrochloride (Tocris 0773) was dissolved in water at a concentration of 50 µg/mL. Three groups of YAC128-55 mice (54–71 days old) were randomly assigned to treatment (n = 6) and control (n = 4) conditions and housed in the PiDose cages. Parameters in the PiDose program were set such that mice in the treatment cohort received two 10 µL drops of memantine solution per gram of bodyweight per day, resulting in a dose of 1 mg/kg of bodyweight per day. Mice in the control group received only water drops. During the first day in the cage, no drug drops were delivered in order to determine a baseline weight for the mice and ensure that mice acquired the operant licking response. Mice remained in the PiDose cages for between 58 and 64 days.

### Statistics

All data analysis and statistics were performed using Python (Python Software Foundation, version 3.7) and Prism 8 (GraphPad Software). Data is expressed as mean ± SEM. Alpha level for all tests was p = 0.05. Pearson correlation coefficients were calculated to measure the linear correlation between PiDose bodyweights and manual bodyweights, and between bodyweight and water consumption. Paired two-tailed t-tests were used to compare total water and memantine consumption between start and end of treatment in the memantine study.

## Results

### PiDose accurately weighs and delivers liquids to group-housed mice

**Figure 2:**
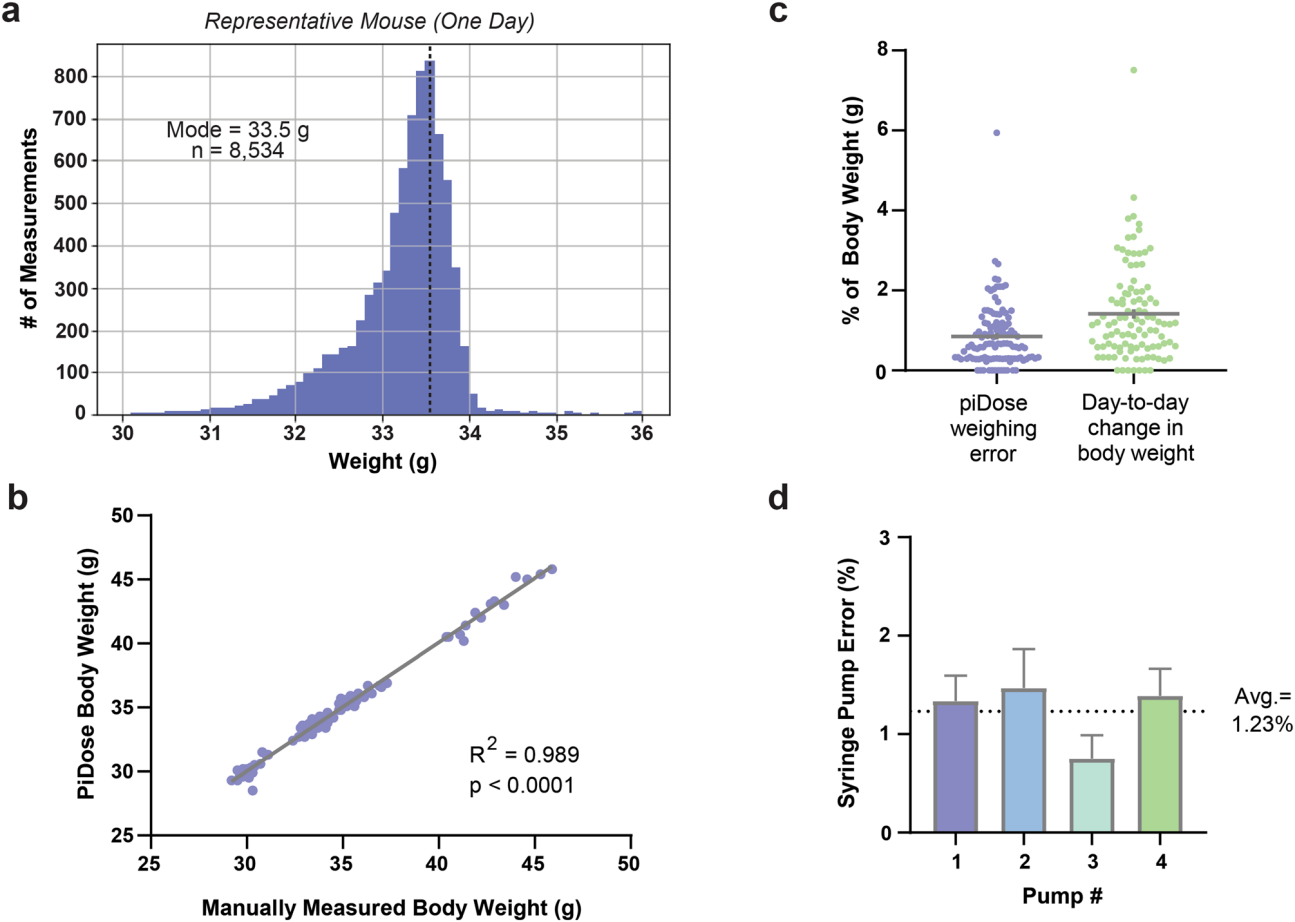
PiDose reliably weighs and administers solutions to group-housed mice. (**a**) Histogram of rounded bodyweight measurements collected for a representative animal over the course of one day. The average bodyweight for the mouse is determined by taking the mode of these values. (**b**) Average daily bodyweights calculated by PiDose are highly correlated with manually measured bodyweights (n = 112). (**c**) The average absolute discrepancy between the PiDose bodyweight and the manual bodyweight (i.e. the weighing error) (n = 112) is smaller than the average day-to-day change in bodyweight (n = 104) for the same animals. (**d**) Average error for each of the four syringe pumps constructed to administer drug-solutions to mice in PiDose expressed as a percentage of the desired drop volume (10 μL). Error bars represent SEM.

In order to assess the accuracy and functioning of different components of PiDose, two cages of wildtype mice (n = 8) were used to test the system. Following RFID capsule implantation, mice were placed in the PiDose cages and allowed free access to the dosing module for a 14-day period. For this initial group, no drug treatment was used, and mice obtained only water from the spout. Within 24 h, all animals learned to lick the spout to trigger water delivery, receiving an average of 1.68 mL of water (± 0.06, n = 112 mouse-days) per day over the test period. An average of 8,820 weight readings (± 612, n = 112), corresponding to ~ 30 min in the dosing module, were obtained per mouse per day. Based on these load-cell measurements, an average daily weight for each mouse was calculated by PiDose (Fig. 2a). In order to determine the accuracy of these bodyweights, animals were also manually weighed daily at midday. There was a high correlation between the PiDose-calculated daily bodyweights and manually obtained bodyweights (R^2^ = 0.989, p < 0.0001, n = 112) (Fig. 2b), and the average absolute discrepancy between the two weighing methods was 0.292 g (± 0.025, n = 112). Expressed as a percentage of bodyweight for each mouse, this translates to an average weighing error of less than 1% (0.853% ± 0.077, n = 112), and is smaller than the average day-to-day change in bodyweight observed over this same period (1.414% ± 0.112, n = 104) (Fig. 2c).

As it is critical that the volumes of drug delivered using PiDose are accurate and consistent, we used 3D-printed syringe pumps for each cage based on the design described by Wijnen et al. (2014)^30^. After determining the number of motor steps required to deliver a drop of approximately 10 µL from a 30 mL syringe, we assessed the accuracy of test drops delivered by these pumps. The average drop size across the four pumps was 9.994 µL (range = 9.723–10.30, n = 36 tests), with an average absolute error of only 0.123 µL (± 0.015, n = 36), or 1.23% of the drop volume (Fig. 2d). Together, these results indicate that PiDose is capable of accurately weighing and delivering solutions to group-housed mice.

### PiDose maintains stable drug treatment over long periods despite day-to-day changes in bodyweight and water consumption

**Figure 3:**
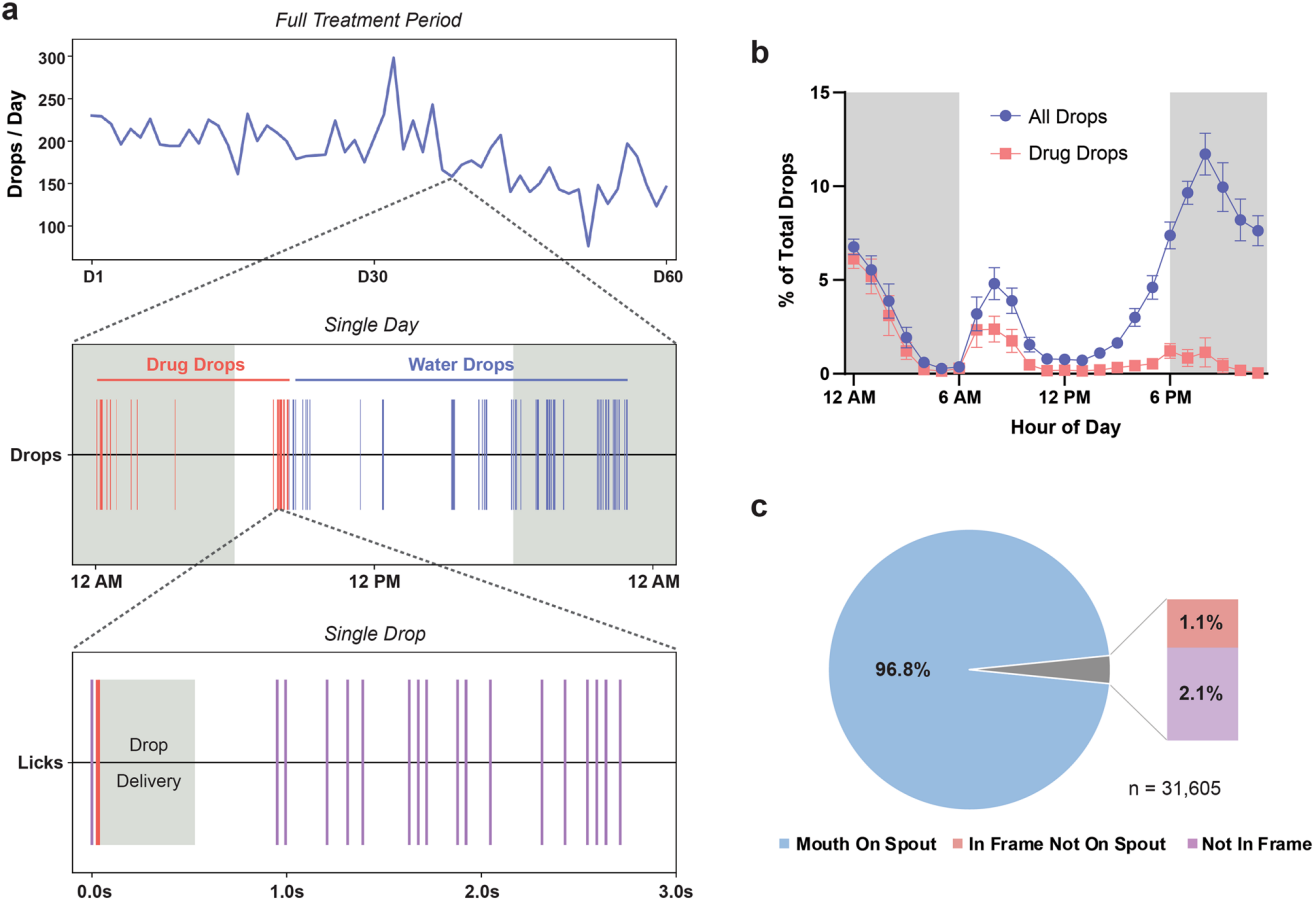
PiDose effectively administers drug to mice over a 2-month period. (**a**) Analysis of the temporal structure of water and drug consumption is shown at several timescales for a representative YAC128 mouse. (**b**) Average total water (n = 10 mice) and memantine solution (n = 6 mice) consumed per hour throughout the treatment period. Memantine solution is dispensed beginning at midnight, and animals typically consumed the required dosage by 12 PM. Mice show clear circadian rhythmicity in drinking behaviour, with most drops dispensed during the dark phase of the light cycle (grey-shaded regions). Error bars represent SEM. (**c**) Analysis of pictures taken immediately after drop delivery confirm that mice are properly triggering and ingesting water and drug solution on the large majority of trials.

We next assessed the ability of this system to treat mice with a fixed drug dosage over an extended period. Memantine is a low-affinity uncompetitive NMDA receptor antagonist that has shown potential as a treatment for Huntington’s disease both in animal models^12,32^ and patients^33^. YAC128 Huntington’s disease mice were assigned to either control or treatment groups and housed in the PiDose cages for two months beginning at 2-months-old. Treatment group mice received 1 mg/kg memantine per day, while control mice received only water. As with wildtype animals, YAC128 mice quickly learned to lick the spout to obtain water and drug solution. The timing of all dosing module entrances, licks and drop deliveries was automatically tracked and recorded by PiDose, allowing for detailed temporal analysis of water and drug consumption (Fig. 3a). Drug administration began at midnight each day and would continue until the mouse had received the required amount of memantine, typically by mid-morning. Overall, a clear circadian rhythmicity was observed, with mice drinking predominantly in the dark phase of the light cycle (73.9% of drops; ± 3.5, n = 10) (Fig. 3b). The average duration of the daily dosing period (i.e. the time from first to last drug solution drop each day) was 10.31 h (± 0.34, n = 375). To confirm that mice were consuming the delivered water and drug solution drops, we positioned a camera adjacent to the spout to take images immediately following drop delivery. Over 30,000 images were taken from the PiDose cages and manually analyzed to assess whether the animal’s mouth was on the spout at the time the drop was dispensed. We found that in 96.8% of images, the animal’s mouth was on the spout immediately following drop delivery, while in the remaining 3.2% the mouse was either not in frame, or in frame but not licking the spout (n = 31,605 total photos) (Fig. 3c).

**Figure 4:**
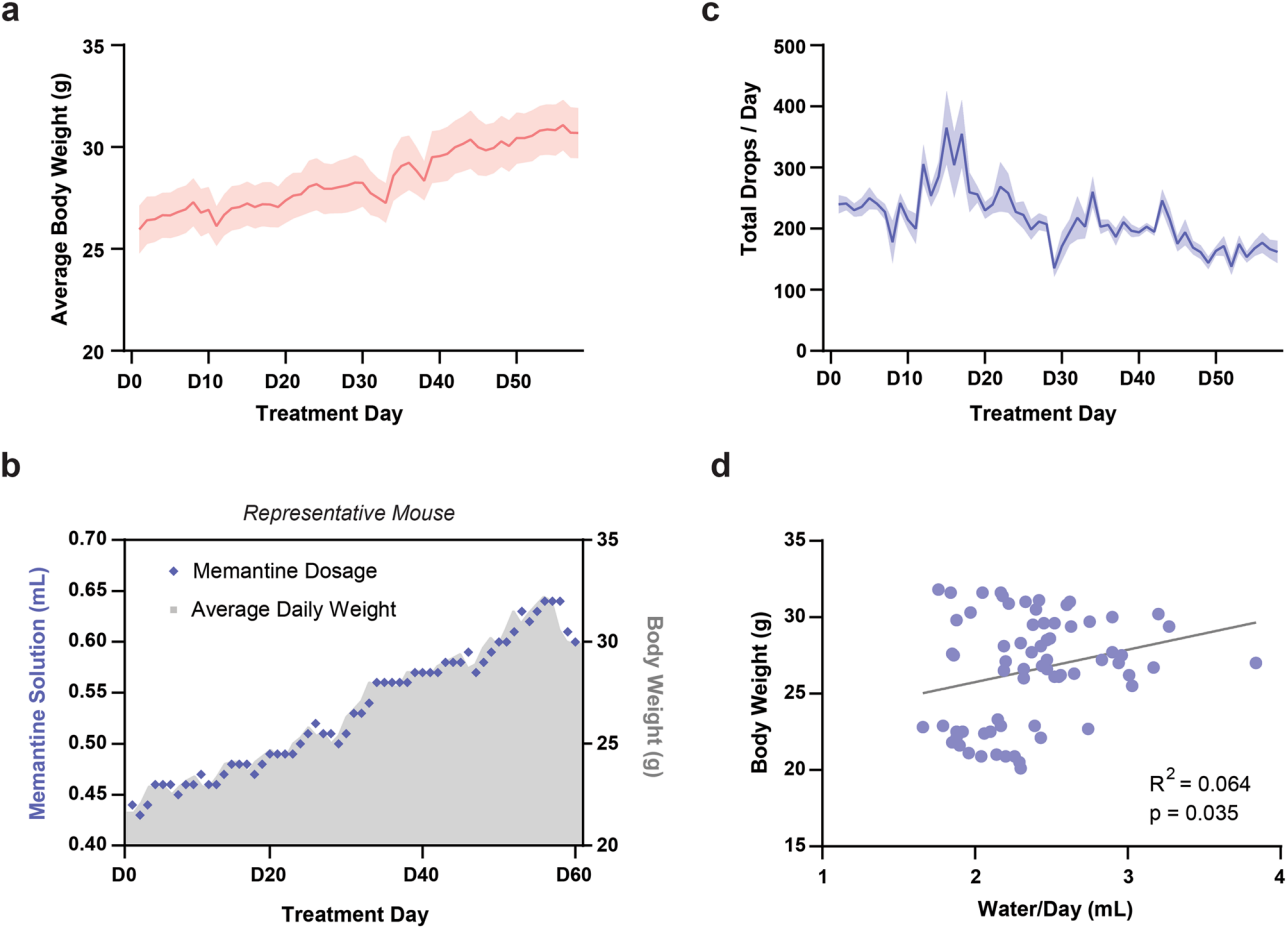
PiDose maintains stable drug treatment despite day-to-day changes in bodyweight and water consumption. (**a**) Mice (n = 10) show gradual and consistent weight gain over the course of two-months of memantine treatment. Data presented as daily mean (red line) ± SEM (red-shaded region). (**b**) The volume of memantine solution dispensed to a representative mouse is automatically adjusted over the course of 2 months to match changes in bodyweight and ensure consistent dosage. (**c**) Mice (n = 10) show substantial day-to-day variability in task engagement and total consumption of drops delivered by PiDose. Data presented as daily mean (blue line) ± SEM (blue-shaded region). (**d**) Daily water consumption during the first week of treatment is positively correlated with daily bodyweight (n = 70), although variability is high.

Over the course of the treatment period mice gradually gained weight, with a ~ 20% increase in bodyweight observed on average (5.12 ± 0.80 g, n = 10 mice) (Fig. 4a). To ensure consistent dosing, PiDose automatically adjusted the amount of memantine dispensed each day, with mice receiving an additional 10 µL drop of memantine solution for every 0.5 g increase in bodyweight (Fig. 4b). In contrast to bodyweight, which changed gradually, the total drops consumed by each mouse varied substantially from day-to-day (Fig. 4c). Interestingly, mice drank less on average by the end of treatment as compared to the beginning despite their increased bodyweight (Week 1 vs. Week 8: t = 5.888, p = 0.0002, n = 10). However, the total number of drops consumed remained consistently higher than the amount needed to receive the required dosage of memantine. As the decrease in water consumption could indicate an effect of PiDose on normal drinking behaviour, we compared daily bodyweight and water consumption in the first week to determine if these measures were correlated at the start of treatment. A positive correlation was observed (R^2^ = 0.064, p = 0.035, n = 70 mouse-days) (Fig. 4d), however the relationship between the two variables was not proportional and the variability was very high.

## Discussion

Reliable drug administration requires that the weight of the animal and the amount of drug being delivered are known with high accuracy. We have demonstrated here that PiDose meets these criteria, exhibiting low weighing error and high delivery accuracy both in terms of measurement of the drug volume and ingestion of the drug. All mice quickly learned to obtain water from the spout, and by training animals to perform an operant response that is identical to and continuous with the act needed to consume the reward we were able to achieve high delivery accuracy. Although we could not confirm that the mouse had consumed the drug on a small percentage (~ 3%) of trials, when combined with the measurement error of the syringe pump this level of precision is comparable to that of manual injection from a 1 mL syringe (tolerance of 5% or more as per the International Organization for Standardization)^34^. We have also demonstrated that PiDose can effectively automate long-term drug treatment. PiDose requires minimal experimenter intervention once running, and the total time required to maintain each system is less than one hour per week. Furthermore, animals can be monitored without the need to enter the facility by connecting to the Raspberry Pi over a secure shell (SSH) network connection and running the system in a compact “headless” configuration without a monitor or keyboard.

In addition to the time-saving benefits of automation, PiDose offers several advantages over existing options in regard to improving the reproducibility of pre-clinical research. First, PiDose involves no handling or direct interaction with animals beyond what is typically required in an animal research facility (e.g. cage cleaning). In contrast, traditional drug administration paradigms often require daily handling and restraint of mice, procedures which are known to cause stress^2,35^. This procedure-associated stress is reported to cause various changes in animal behaviour and physiology, potentially masking or enhancing the effects of drug treatment. For example, handling and/or injection stress has been shown to alter the behavioural response to anxiogenic drugs^36^, increase immobility time in the forced-swim test^37^, activate immediate early gene transcription in stress-responsive brain regions^38^ and alter immune function^39^. Furthermore, the response to treatment-associated stress may vary between genotypes, further complicating the interpretation of results. YAC128 mice, for instance, show a depressive phenotype^40^ and may consequently be more sensitive to the chronic stress of repeated injections^37^. Confounds such as this could introduce systematic error to the outcome measures of drug experiments and obscure treatment effects.

A second advantage of PiDose with regards to reproducibility is that of improved dosing accuracy and consistency when compared to other home-cage methods. Indeed, our data suggests several issues with the commonly used strategy of mixing a drug in directly with the animals’ home-cage drinking water. Although an overall correlation between bodyweight and water consumption was observed in PiDose, the relationship was not proportional (i.e. a 40-g mouse did not drink twice as much as a 20-g mouse). As a result, when the ‘drinking water’ strategy is used in group-housed mice, it is likely that heavier mice in the cage receive a lower dosage on average than lighter mice. This could be especially problematic in mixed genotype cages where average bodyweight, and consequently average drug dosage, varies by genotype. Indeed, this is reported to be the case with heterozygous YAC128 mice who weigh more on average than their wildtype littermates^31,41^. In addition, water consumption in PiDose varied by as much as 30–40% from day-to-day, while bodyweight changed comparatively slowly. As a result, even if mice are single-housed, day-to-day dosage cannot be properly controlled by mixing the drug in directly with the mouse’s drinking water. These inconsistencies could result in substantial differences in the effective dose received by each animal over the course of the treatment period, increasing the inter-animal variability of treatment outcome measures and consequently the risk of Type I and II errors. This dosing error also complicates any conclusions regarding the drug’s dose–response relationship and increases the risk that the treatment will fail to translate to human use.

Although PiDose presents many advantages over alternative methodologies, there are also some important limitations to consider. First, its use is restricted to drugs that can be dissolved in water, are stable in solution and can be kept at room temperature. In addition, the specific timing of drug treatment each day cannot be precisely controlled, as mice have free access to the spout and consume the drug solution in a self-directed manner. For this reason, PiDose may not be appropriate for studies where the drug must be given at a precise time every day, or at specific intervals throughout the day. Nevertheless, the temporal pattern of drug administration can be broadly set by adjusting certain parameters within the software, and by changing the concentration of drug solution. For example, a high drug concentration could be used to shorten the average length of the dosing window and approximate an acute treatment method like oral gavage. This may be useful for treatments where a higher blood concentration of the drug is required in order to elicit effects. On the other hand, for rapidly metabolized compounds, it might be preferable to administer the drug throughout the day. For this purpose, a parameter in the PiDose program can be set so that the system dispenses a drug drop only once every two or three drops. With some minor modifications, it should even be possible to calculate a theoretical blood concentration of the drug for each animal at any given time based on their bodyweight, the number and timing of previous drops, and known metabolic characteristics of the drug. This information could then be used to determine whether to deliver a drug or water drop in response to a lick, with the goal to keep the blood concentration of the drug within a target range.

Our use of PiDose has so far been restricted to FVB/NJ strain male mice of 2- to 4-months-old; however, we expect that both male and female mice of a range of ages and different strains could be treated using this system. For the treatment of very small or very large animals, some modifications to the dimensions of the dosing module may be necessary to ensure that only one mouse can enter at a time. Nevertheless, we found that our configuration worked well for mice varying in bodyweight from 20 to 45 g. The load cell used with PiDose has a maximum capacity of 780 g (including the weight of the dosing module), which should be more than sufficient for any mouse applications and could even work in a modified system for treating larger rodents (e.g. rats). We found that the load cell maintained accurate bodyweight measurements across the range of animal sizes assessed without the need for recalibration. All other sensors and electronic components should be compatible with physical modifications of the dosing module, provided that the RFID reader is positioned such that it will come into close proximity with the animal’s RFID tag. In order to facilitate any adjustments that other users may want to make, we have provided the original design files for all 3D parts online (https://osf.io/rpyfm/).

The potential of open-source tools in biological research has gathered attention as of late^42^, and it is our hope that by making the code and design files for PiDose open-source and freely accessible, others will adapt and improve it as necessary for their use. Although PiDose does require some wiring and basic tools to assemble, the skills required are straightforward and can be quickly learned. Assembling a full PiDose system costs only ~ $300 and requires no more than a few days. Given the now well-established concerns regarding the reproducibility of many pre-clinical studies^43,44^, the need for a tool that can increase the accuracy of drug dosing while also decreasing the exposure of animals to stressful stimuli is critical. We believe that PiDose holds promise in this regard and should prove useful for both basic and pre-clinical biological research.

## Supplementary information

Supplementary Information 1.

Supplementary Information 2.

## Data Availability

The datasets generated during and/or analysed during the current study are available from the corresponding author upon reasonable request.
