# Supplementary material for: PiDose: an open-source system for accurate and automated oral drug administration to group-housed mice
Source: Sci Rep. 2020 Jul 14;10:11584. doi: 10.1038/s41598-020-68477-2 (PMC7360602; doi:10.1038/s41598-020-68477-2)
Supplement: Supplementary file 1 — Supplementary Information 1. [file 41598_2020_68477_MOESM1_ESM.docx]

**PiDose: An open-source system for accurate and automated oral drug administration to group-housed mice**

Cameron L. Woodard, Wissam B. Nasrallah, Bahram V. Samiei, Timothy H. Murphy and Lynn A. Raymond

Supplementary Information

**Supplementary Table S1: Parts List**

| **Component** | **Amount** | **Supplier** | **Part Number** |
| --- | --- | --- | --- |
| PLA Filament, 1.75mm Black | 250 grams | MakerBot | MP05775 |
| Raspberry Pi 3B | 1 | Newark | RASPBERRYPI3-MODB-1GB |
| 16GB Micro-SD Card | 1 | Verbatim | 44082 |
| Pi Cobbler | 1 | Adafruit | 2029 |
| Female Headers | 3 | Adafruit | 598 |
| Perma-Proto Board | 1 | Adafruit | 1606 |
| L293D Motor Driver | 1 | Adafruit | 807 |
| Solenoid Valve | 1 | Gems Sensors | MB202-VB30-L203 |
| RFID Reader ID20-LA | 1 | Sparkfun | SEN-11828 |
| RFID Reader Breakout | 1 | Sparkfun | SEN-13030 |
| 2mm 10-Pin Socket | 2 | Sparkfun | PRT-08272 |
| RFID Glass Capsules | 1 | Sparkfun | SEN-09416 |
| Load Cell Amplifier | 1 | Sparkfun | SEN-13879 |
| EasyDriver Stepper Motor Driver | 1 | Sparkfun | ROB-12779 |
| 0.78kg Micro Load Cell | 1 | Phidgets | 3132 |
| Capacitive Touch Sensor Breakout | 1 | Adafruit | 1982 |
| RPi Camera (F) | 1 | Waveshare | 10299 |
| Raspberry Pi Camera Flex Cable (18") | 1 | Adafruit | 1730 |
| 6" Female-Male Jumper Wires | 17 | Adafruit | 826 |
| 2.2kΩ Resistor | 2 | Adafruit | 2782 |
| 5V 2.5A Power Supply | 1 | Adafruit | 1995 |
| 12V 1A Power Supply | 1 | Adafruit | 798 |
| In-Line Barrel Jack Power Switch | 1 | Adafruit | 1125 |
| Female DC Power to Screw Terminal | 1 | Adafruit | 368 |
| 32 Oz Bottle w/Tubulation | 1 | US Plastic | 68024 |
| 1/4" Beverage Tubing | 0.5 m | US Plastic | 54418 |
| 1/4" to 1/8" Tubing Coupler | 1 | US Plastic | 64896 |
| 1/8" Beverage Tubing | 1-2 m | US Plastic | 54413 |
| 1/8" Luer | 2 | US Plastic | 67158 |
| 3/16" Tubing Clamp | 1 | US Plastic | 16004 |
| 30mL Luer-Lock Syringe | 1 | BD | 309650 |
| 18G Needle | 2 | BD | 305195 |
| 2" Aluminum Spacers | 6 | Siskiyou | AS-2.00 |
| 6mm Polycarbonate Sheet | 30x45 cm | Robertson Plastics | GPC-BS903CL0250 |
| M2x6 Machine Screw (Phillips) | 6 | Newark | 53M8647 |
| M2x12 Machine Screw (Phillips) | 2 | Newark | 53M8640 |
| M3x8 Machine Screw (Hex Head) | 6 | Newark | 53M8806 |
| 1/4"-20 Cap Screw, 5/8" Long (Hex Head) | 7 | Thorlabs | SH25S063 |
| 1/4"-20 Set Screw, 1/2" Long | 6 | Thorlabs | SS25S050 |

**Supplementary Data 1: PiDose 3D Files**

Contained in this ZIP archive are six STL files for 3D-printing the components of PiDose. Two additional pieces are saved as SVG files and must be laser-cut from acrylic. The nose_poke_piece file should be laser-cut from 3mm clear acrylic and the spout_window piece from 2mm clear acrylic.
